# Eugenol Nanoparticles in Dental Composites: Literature Review of Antimicrobial, Anti-Inflammatory, and Clinical Applications

**DOI:** 10.3390/microorganisms13051148

**Published:** 2025-05-16

**Authors:** Fatima Zahra Kamal, Alin Ciobica, Gabriel Dascalescu, Said Rammali, Mohamed El Aalaoui, Radu Lefter, Ioana Vata, Vasile Burlui, Bogdan Novac

**Affiliations:** 1 Higher Institute of Nursing Professions and Health Technical (ISPITS), Marrakech 40000, Morocco; fatimzahra.kamal@gmail.com; 2 Department of Biology, Faculty of Biology, Alexandru Ioan Cuza University of Iasi, 20th Carol I Avenue, 700506 Iasi, Romania; gabidascalescu2001@gmail.com; 3 Center of Biomedical Research, Romanian Academy, 700506 Iasi, Romania; radu_lefter@yahoo.com; 4 Clinical Department, Apollonia University, Păcurari Street 11, 700511 Iasi, Romania; secretariat@univapollonia.ro; 5 CENEMED Platform for Interdisciplinary Research, “Grigore T. Popa” University of Medicine and Pharmacy of Iasi, 16th Universitatii Street, 700115 Iasi, Romania; 6 Laboratory of Agro-Alimentary and Health, Faculty of Sciences and Techniques, Hassan First University of Settat, BP 539, Settat 26000, Morocco; said.rammali90@gmail.com; 7 Human Nutrition, Bioacives and Oncogenetics Team, Faculty of Sciences, Moulay Ismail University, Meknes 11201, Morocco; 8 National Institute of Agricultural Research, Avenue Ennasr, BP 415 Rabat Principal, Rabat 10090, Morocco; mohamed.elaalaoui@inra.ma; 9 Faculty of Medicine, “Grigore T. Popa” University of Medicine and Pharmacy of Iasi, 16th Universitatii Street, 700115 Iasi, Romania

**Keywords:** antibacterial, anti-inflammatory, biofilm, control release, eugenol, restorative dentistry

## Abstract

The formation of microbial colonies and biofilms are common on dental restorations. This can lead to secondary caries. Another common complication is the post-operative inflammation noted in patients. The traditionally used dental composites are designed without the inherent components having antimicrobial and inflammatory properties. This has become a major challenge in current restorative dentistry applications. In order to address these challenges, a possible approach is to incorporate eugenol nanoparticles (NPs) into dental composites. This approach can offer dual therapeutic benefits since eugenol possess both antimicrobial and inflammatory properties. In fact, compared to synthetic antimicrobial agents, eugenol exhibits antibacterial activity not only against *Streptococcus mutans* but also against a range of oral pathogens. It also exhibits anti-inflammatory effects that can promote healing by reducing post-operative sensitivity. In spite of the above benefits, eugenol cannot be incorporated directly into dental materials. This is because eugenol is highly volatile and has poor water solubility. The encapsulation of eugenol in suitable nano-materials can overcome these limitations. In addition, it can enable the controlled and sustained release of desirable agents for long-term therapeutic action. In this review, we explore the mechanisms, advantages and potential clinical applications of dental composites containing NP integrated with eugenol. We highlight the advantages of having antimicrobial and anti-inflammatory functions in a single restorative material. At the same time, we acknowledge the need for more in-depth research to optimize NP formulations with eugenol that does not compromise the mechanical properties of dental materials. Based on a thorough literature review, we believe that this approach has much potential in restorative dentistry procedures that will aid therapeutic outcomes in the future.

## 1. Introduction

Dental caries is among the most common diseases experienced by people worldwide. It is also among the most easily preventable diseases. Dental caries affects almost everyone at some point in time, differing only in severity. The disease can range from minor spots on the enamel to extensive decay that reaches the root canal [1]. According to the World Health Organization’s (WHO) Global Oral Health Status Report (2022), dental carries affects approximately 2 billion adults and 514 million children [2]. A systematic review published earlier in 2020 represents these data in relative terms and estimates that dental caries affects 46.2% of deciduous teeth (in children) and 53.8% of permanent teeth (in adults) throughout the world [3]. Some studies also report regional disparities in populations with lower prevalence rates observed in European populations compared to the higher rates found in African communities [4]. Also, an estimated 1.3 to 1.6 million Years Lived with Disability (YLD) are attributed to caries of deciduous and permanent teeth, respectively [5]. A study by Meier et al. (2017) took into account the prevalence of oral disease burden (due to sugar intake) in 168 countries, and reported that the annual treatment costs exceed USD 172 billion [6]. Overall, these data clearly suggest the economic and social burdens imposed due to dental caries worldwide.

Secondary caries is one of the most common reasons for restoration replacement and forms at the existing restoration margins [7]. It is located gingivally, regardless of the type of restoration or material [8]. In contrast to primary caries, the term secondary caries refers to caries occurring after a filling has been placed on a cavity for some time, usually because of micro-cracking greater than 50 μm, which permits bacterial infiltration [9]. However, the connection between visible gaps and secondary caries is weak, almost meaning that secondary caries is an isolated event occurring due to certain conditions promoting the formation of cariogenic plaque [10]. From a clinical standpoint, secondary caries is difficult to diagnose when the consistency of dentin and enamel becomes firmer and more discolored and thus changes in color [7]. Prevention includes the management of micro-cracks, the application of fluoride, proper oral hygiene, and regular check-ups [9]. Restoration evaluation and replacement should follow a conservative approach [10].

The occurrence of secondary caries further exacerbates the dental disease burden. Secondary caries is an infection that occurs at the margins of dental restorations. It compromises the outcomes of dental treatments and necessitates repeated interventions. In practice, the composite as well as amalgam restorations are susceptible to secondary caries [11,12]. In spite of the significant disease burden of secondary caries indicated in the literature, accurate global statistics are not reported by the WHO. However, many regional studies confirm its high occurrence. These studies also suggest that compromised oral hygiene and affected gingival margins are good predictors of secondary caries [13]. In a study from Saudi Arabia, Alenezi et al. (2022) reported that secondary caries affected 4% of patients with good oral hygiene, 5.5% with fair oral hygiene, and 18.4% with poor oral hygiene [14]. Nedeljkovic et al., 2020, reported an overall prevalence of 3.6% in a Dentistry University in Belgium, with 72% of diagnosed cases having inflamed gingival margins that required restoration replacements [12].

The literature suggests the co-existence of over 700 microbial species in the oral cavity under normal oral hygiene conditions. These microorganisms maintain a symbiotic balance and prevent the overgrowth of any single microbial species [15]. This is possible because regular brushing techniques and oral hygiene measures prevent formation of biofilms and the overgrowth of pathogenic bacteria [16]. However, in the case of dental restorations, the adhesive interface between the tooth structure and restorative materials is susceptible to degradation. This can lead to bacterial infiltrations at the degraded sites [17]. Eventually, the infiltrated bacterial flora can form biofilms on the restorative surfaces, making the material surfaces rough in texture. In turn, this activity further promotes bacterial colonization and plaque accumulation on the roughened surfaces [18]. *Streptococcus mutans* is the main etiological agent of primary as well as secondary caries followed closely by Lactobacillus species. These bacteria can even affect people maintaining good oral hygiene. This is because the interfaces between the tooth structure and restorative materials are highly uneven and remain inaccessible to standard brushing, cleaning, and oral hygiene measures. Thus, there remain microenvironments in the form of small gaps, ditches, and micro-cracks, in and around the dental restoration materials where bacteria and other microbial pathogens can infiltrate, multiply, and accumulate [11,12]. The improper isolation and inoculation techniques used during the restorative procedures also risk bacterial contamination [18].

The conventionally used dental composites have been immensely useful in the restoration of carious lesions. However, they also carry a substantial risk of the occurrence of secondary caries [19,20]. Primarily, the dental composites were designed to provide esthetic appeal and mechanical support to the dental structure. Hence, they lacked intrinsic antibacterial and anti-inflammatory properties. Over time, it has been realized that the original design of dental composites does not adhere completely to the enamel structures, leaving micro-spaces. In the absence of antibacterial agents, this increases the susceptibility of restorative interfaces to bacterial infiltrations which eventually facilitates colonization by cariogenic microorganisms [17]. More recently, many antibacterial dental composites have been developed to address this limitation. However, they commonly fail to provide sustained therapeutic effects in dental materials such as restorations, implants, or orthodontic brackets. As a result, they do not provide the desired effects of long-term application and acting against the robust survival mechanisms of bacterial biofilms [21]. Another challenge faced with the incorporation of synthetic antibacterial additives is their concentration-dependent activity. In the absence of sustained release mechanisms, higher concentrations of antibacterial agents are released in the oral cavity. This can lead to cytotoxicity [22]. In the literature, many of the commonly used unpolymerized monomers in dental composites are linked with cytotoxic activity. A few examples include Bis-GMA, UDMA, HEMA (2-hydroxyethyl methacrylate), TEGDMA, camphorquinone, and glutaraldehyde [23,24,25]. There are also reports of limitations that can arise due to the compromised physical properties or esthetic appeal (loss of intended color) of restorative materials upon combining them with some antibacterial agents [26]. Overall, these limitations challenge the development of a standardized design for dental materials with antibacterial and anti-inflammatory properties without compromising their mechanical and chemical stability.

The present review provides an overview of the design and potential advantages of an innovative dental composite that integrates eugenol derivatives in NPs. This review primarily focuses on the literature supporting the controlled release mechanisms that are necessary to ensure sustained antibacterial and anti-inflammatory effects in the dental composite model. Additionally, it explores and highlights the limitations of existing antibacterial restorative materials and further proposes solutions that can enhance patient outcomes.

Our key objectives are as follows:

- Surveying the evidence on the dual therapeutic properties and accompanying limitations of eugenol.
- Exploring nano-encapsulation as a strategy to preserve both therapeutic and mechanical functions.
- Highlighting the clinical implications for high-risk groups with recurrent caries or periodontal disease.
- Filling gaps and structurally guiding the orthogonal research direction on formulating more effective NPs.

## 2. Methodology

An extensive literature review was carried out, and specific studies concerned with the properties and uses of eugenol in restorative dentistry were revealed. The literature review included articles published from January 2010 to December 2023. Databases searched included PubMed, Scopus, and Web of Science. The search strategy involved combinations of the keywords: Eugenol, antibacterial, anti-inflammatory, controlled release, biofilm, and restorative dentistry, utilizing Boolean operators such as AND and OR. Only peer-reviewed English-language articles were included. Titles and abstracts were screened after the deletion of duplicates, followed by full-text evaluation to ensure the studies’ inclusion with relevant data on the topic.

## 3. Problem Addressed

### 3.1. Caries Recurrence and Secondary Infections

Dental caries is a complex disease etiology and involves an intricate interplay of multiple factors. Even the simplest description of dental caries recognizes it as a multifaceted biofilm-mediated, sugar-driven, and behavior-modifiable disease [27]. Basically, the disease occurs as a result of diverse metabolic activities of acid-producing microorganisms in the oral cavity and the end-products of fermentable carbohydrate residues left on tooth structures that they utilize as food for growth and multiplication [1]. In addition, the unique determinants of an individual, such as their genetics, tooth mineralization status, saliva composition, nutrition and food choices, immune responses, and oral hygiene practices, contribute to the complexity of dental caries [1,27]. Acknowledging this complex etiology makes it easier to comprehend the higher rates associated with failure in dental restoration procedures. Practically, the bond strength is significantly compromised when new restorative materials are placed over the existing ones or unprepared tooth structures. Consequently, the improper dental restorations lead to patients requiring re-restoration interventions [28]. If the restoration margins are inadequately sealed, they further compromise the efficacy of restorative materials by providing niches for bacterial infiltration and growth due to microleakage [17]. Another reason for failed interventions over time includes the improper excavation of initial carious lesions. In addition, factors such as low-quality restorative materials, restoration class, and smoking habits form the key risk factors for failed restorative interventions [12].

Literary evidence suggests that restorations fail in over 50% of cases due to the development of secondary caries [19,20]. *S. mutans* and *Lactobacillus* species play a crucial role in cariogenesis (progression of tooth decay) by fermenting dietary sugars into organic acids (primarily lactic acid). They also demineralize the enamel and dentin in the process. The prolonged acidic exposure due to the activities of the primary etiological agents shifts the microbial equilibrium in the oral cavity. It begins with the suppression of acid-sensitive bacteria in the oral cavity and forms an environment that supports the growth of acid-tolerant secondary colonizers. In later stages, these pathogens thrive and produce protective biofilms [12,19]. Once established, these bacterial biofilms contribute to the progression of secondary caries and jeopardize the longevity of the restorations.

Overall, the combination of poor marginal sealing, bacterial biofilm formation, and the absence of sustained antibacterial action in existing restorations has been widely recognized as a fundamental limitation in restorative dentistry.

One of the most common reasons for restoration failure is secondary caries, which can be affected by many factors including patient caries risk, restoration gaps, and the experience of the dentist [29]. Prevention for secondary caries is similar to primary caries, as it relies heavily on fluoride, dietary changes, and oral hygiene [30]. Patients must keep attending regular dental checkups and receiving professional cleaning to increase the rate of early detection of marginal breakdown or recurrent decay before it progresses [31]. Composite restorations seem to be more likely than amalgam restorations to have secondary caries, especially in patients at a high caries risk [30]. The secondary caries prevalence varies in reporting, with one study citing it as being as high as 26% in patient populations [32], with a substantial amount occurring during MOD restorations and composite fillings [32]. The use of mouthwash was positively correlated with lower rates of secondary caries [32]. Current methods for detecting secondary caries lack any empirical research or validation, and there is a possibility that they lead to over-detection [29]. The secondary caries management strategies include repairing the defective part, or replacing the restoration in its entirety [29].

Research regarding materials that can prevent the secondary caries appearance has explored strategies. The resin-based composites inserted with antibacterial agents such as quaternary ammonium compounds and chlorhexidine have revealed promise in inhibiting bacterial growth [33]. Fluoride-releasing materials, including glass ionomers and fluoride-containing amalgams, can prevent caries by positively influencing re-mineralization and inhibiting bacterial activity [34]. Bioactive composites with amorphous calcium phosphate or bioactive glass can release calcium, and phosphate ions can contribute to re-mineralization and restoring the dentin bond [35]. In vitro studies have indicated that fluoride-releasing materials inhibited the demineralization of enamel and dentin; however, clinical evidence concerning their effectiveness on inhibiting secondary caries remains inconsistent and inconclusive [36]. The development of these materials often requires the balance between mechanical properties and bioactivity to ensure optimal performance in dental restorations [35].

### 3.2. Inflammation and Tissue Irritation

Dental tissues are uniquely organized compared to other tissues in the human body. Teeth are complicated structures made up of several distinct tissues with unique properties and functions. This complex structure allows the tooth to serve its essential functions in the overall process of mastication and articulation, as well as maintaining the ability to respond to challenges presented by the environment [37]. The crown is covered by enamel, the hardest and most mineralized tissue in the human body, consisting of about 96% inorganic material [38]. Dentin, the tooth bulk, is located beneath the enamel and is approximately 70% mineralized [39]. The root is covered by cementum, which connects to the alveolar bone through the periodontal ligament (32). At the tooth center is the pulp, containing nerves, connective tissue, and blood vessels [37]. Dentin and cementum both contain organic material (mainly collagen) while enamel is primarily inorganic [40].

In this structure, the soft tissues known as the dental pulp are encapsulated within the enamel and dentin, which are rigid mineralized structures. The presence of dense neurovascular network in the dental pulp makes them very sensitive. Further, these networks play an important role in regulating inflammatory mediators. Overall, this structure and organization of the dental tissues make them highly responsive to external stimuli, which makes the rapid triggering of the body’s immune defense mechanisms possible [41]. In practice, the restorative dental procedures cause physical trauma to the dental pulp due to the use of mechanical or chemical irritants. This region is also exposed to a range of microorganisms from the surrounding tissues in the oral cavity. In addition, in rare cases, the instruments used for such procedures can introduce environmental flora in the dental cavity. In particular, the deep cavity preparations are highly critical and labor-intensive, and can provoke an inflammatory response even upon use of very precise techniques in the pulp and surrounding tissues [42].

A search of the literature highlights numerous inflammatory mediators that are involved in the progression of dental pulp inflammations [43]. A few examples of these inflammatory mediators include pro-inflammatory cytokines [Interleukin (IL)-1, IL-6, IL-12, IL-18, tumor necrosis factor (TNF)-α, and interferon (IFN)-γ], anti-inflammatory cytokines (IL-4, IL-5, IL-10, and IL-13), chemokines, nitric oxides, proteases, eicosanoids, and neuropeptides. The pro-inflammatory cytokines amplify immune responses and contribute to tissue destruction. The anti-inflammatory cytokines modulate the immune response and prevent excessive tissue damage, whereas chemokines, described as signaling proteins, attract immune cells to the inflamed sites. The nitric oxide and proteases are involved in the vascular regulation and degradation of extracellular matrix, respectively. The lipid mediators such as eicosanoids are mediators of inflammatory signaling and pain perception. The neuropeptides are compounds which are released from the peripheral nerve terminals. These peptides are also mediators of inflammatory responses in the dental pulp. A few examples of neuropeptides include calcitonin gene-related peptide, Substance P (SP), Neurokinin A, Vasoactive intestinal peptide, and Neuropeptide Y [43,44]. Specifically, a study linked deep cavity preparations with an increased release of SP, which causes neurogenic inflammations [45].

A number of drawbacks associated with tissue inflammation and irritation are part of the currently practiced restorative procedures in dentistry. Hence, advancements are continuously sought by integrating materials into the dental restorations that fulfill the property of healing and tissue regeneration. Among the materials tested and used so far, the Glass Ionomer Cement (GIC) and Calcium Silicate-Based Materials such as Mineral Trioxide Aggregate—MTA are the most notable. The GIC has fluoride-releasing capabilities, which are combined with bioactive glass or calcium orthophosphate particles (to improve stability) and used to promote the re-mineralization of the soft dentin [46]. The MTA, composed of calcium oxide (50–75%) and silicon dioxide (15–25%), is another example of a biocompatible and bioactive material which is commonly used in pulp capping and root repair applications. It facilitates the dentin bridge formation and supports pulp healing [47]. However, studies have associated the use of these compounds with an increase in inflammatory responses. For instance, a study evaluated the biocompatibility of resin-modified GIC and reported that the application of this material in deep cavities with minimal dentin thickness caused a significant inward diffusion through dentinal tubules. This diffusion occurred as a result of the disrupted odontoblastic layer and resulted in a moderate to intense inflammatory response in the pulp tissue [48]. In another study, Tabari et al. (2020) reported the occurrence of a moderate-to-severe inflammatory response on the 7th day after the implantation of MTA mixed with calcium lactate gluconate [49]. The commonly used resin-based composites bonded to enamel are also frequently associated with higher inflammatory responses [50,51]. In addition, an early study on inflammatory responses to dental composites in rat models demonstrated the development of lung diseases and chronic inflammatory responses triggered by the grinding and polishing of plastic-based dental composites. The vacuoles within alveolar macrophages and the interstitium showed the presence of 0.5–10 micron-sized particles. The study also reported the presence of chronically inflamed foci around restorative dental material [52].

The absence of mechanisms to prevent infection and inflammation in many restorative materials currently used in dental procedures represents a significant limitation in restorative dentistry. These factors are responsible for rapid tissue degeneration and necrosis, which, if left unchecked, can spread to surrounding periodontal structures and lead to irreversible damage [41,42].

### 3.3. Limitations of Existing Antibacterial Approaches

The synthetic antibacterial agents in use have been investigated for their compatibility with dental composites. While these materials show good efficacy on initial application, the rapid release kinetics exhibited within leads to a decline in antibacterial efficacy over time [53]. This further increases the concerns regarding cytotoxicity and the potential development of bacterial resistance. The resulting negative environmental impact of these materials limits their widespread adoption [32,54]. The resin-based dental composites, in general, are believed to be highly susceptible to bacterial infiltrations, and they are more commonly associated with recurrent caries [12,55,56].

Silver nanoparticles (AgNPs) have also gained significant attention in dental applications due to their durability and remarkable antimicrobial properties. AgNPs effectively combat bacterial and fungal infections associated with dental plaques. Hence, they are a popular choice in dental restoration materials, including composite resins, denture bases, adhesives, and implants [57]. However, despite their efficacy, AgNPs also raise concerns regarding their potential toxicity. Particularly, this is due to the ability of AgNPs to penetrate biological barriers and accumulate in vital organs, including the lungs, kidneys, liver, spleen, and testes [58]. AgNPs can also cross the blood–brain barrier and accumulate in brain tissues. This is highly concerning, since it leads to neuronal and glial cell dysfunction, causing neurotoxic effects [58,59]. Given the prolonged exposure to AgNPs in dental restoration materials, especially implants and denture bases, there is an increased risk of these particles entering the bloodstream either through mucosal absorption or ingestion [60].

Among early studies, a systematic review conducted up to July 2013 identified no Randomized Controlled Trials (RCTs) that compared antibacterial dental composites with those without antibacterial activity [61]. To the best of our knowledge, no RCTs have compared the same up till now. In general, several studies have attempted to design restorative materials with antimicrobial properties. However, they are met with several challenges [26]. More commonly, the antimicrobial agents are influenced by environmental factors such as saliva composition and pH fluctuations that lead to a decrease in their activity. In some cases, they also compromise the physical properties of the restorative materials. The activity of bioactive compounds can also be affected due to the mechanical wear of the restorative material from chewing [32,62]. The esthetic appeal (change in color) of restorative materials is also compromised in many cases due to bio-chemical factors [63]. Some studies have also described an alteration in the adhesive property of restorations to the tooth due to decreased bond strength [64]. As the antibacterial agent depletes or becomes inactivated due to any of the above reasons, the risk of pathogen re-colonization increases. This potentially compromises the long-term success of the restoration materials. Another challenge with incorporating synthetic antibacterial additives is their concentration-dependent activity. In the absence of a sustained release mechanism, higher contents of antibacterial agents may cause cytotoxicity or interfere with the mechanical properties of the composite [26]. There are also reports where none of the chemical or mechanical properties of restorative materials are compromised, but the concentrated broad-spectrum antibacterial agents significantly disrupt the oral microbiome balance [32,65,66,67,68,69]. Such disruptions lead to secondary infections or other oral health complications. Consequently, most antibacterial agents are added in small amounts and modified to have significant antibacterial effects, minimal cytotoxicity, and no effects on the mechanical characteristics of the dental composites. Even the most recent review published in 2024 reported the lack of a standardized design for dental materials with antibacterial properties that does not compromise their mechanical and chemical stability [22].

Besides the technical, mechanical, physical, and esthetic limitations, a major drawback of antibacterial dental materials is their narrow-spectrum activity. They are primarily designed and tested against *S. mutans*. While *S. mutans* is a key contributor to dental caries, oral infections are inherently polymicrobial [15,16]. The pathogenicity of *S. mutans* is not solely responsible for disease progression; rather, it is amplified through interactions with other microorganisms such as *Candida albicans*, *Enterococcus faecalis*, *Propionibacterium acnes*, and *Actinomyces naeslundii* [12,19]. These microbes, either individually or in combination, contribute to the formation of biofilms, which significantly enhance their resilience against antibacterial treatments. In this scenario, we are presented with a critical challenge of failed antibacterial strategies due to the ineffective penetration or disruption of these biofilms. As a result, even if a restoration material initially suppresses bacterial growth, the residual surviving microorganisms form new biofilms and eventually lead to re-colonization. Ultimately, this reduces the long-term efficacy of the antibacterial intervention [15,16].

Dental biofilm formation is a multifaceted process and involves many stages and interactions among bacteria. Initially, the biofilm formation starts with the aggregation of early colonizers to salivary pellicle proteins on tooth surfaces [70]. As the biofilm develops, there is a coaggregation between the previously established species and the new colonizers, allowing for increased diversity of the species in the developing biofilm [71]. The maturing biofilm exhibits spatial organization and stratification, owing to metabolic synergies and interactions between species [72]. These interactions include physical cell–cell associations, a form of interspecies signaling, and the production of antimicrobial compounds [72,73]. Within a dental biofilm, bacteria cooperate to metabolize complex molecules and perform various functions while simultaneously competing through various mechanisms, like the production of bacteriocins and quorum sensing [71,73]. Understanding these complex interactions and biofilm development is critical in developing effective methods of prevention or treatment for oral conditions including periodontitis and dental caries [70,72]. While more sophisticated approaches, such as targeting specific virulent genes or employing quorum quenching in restoration materials, can enhance antibacterial activity, they also introduce new challenges [74]. The stability of these agents, their sustained activity in the oral environment, and their safety over prolonged periods are critical factors that require further investigations.

## 4. Innovative Design

### 4.1. Why Eugenol?

**Figure 1:**
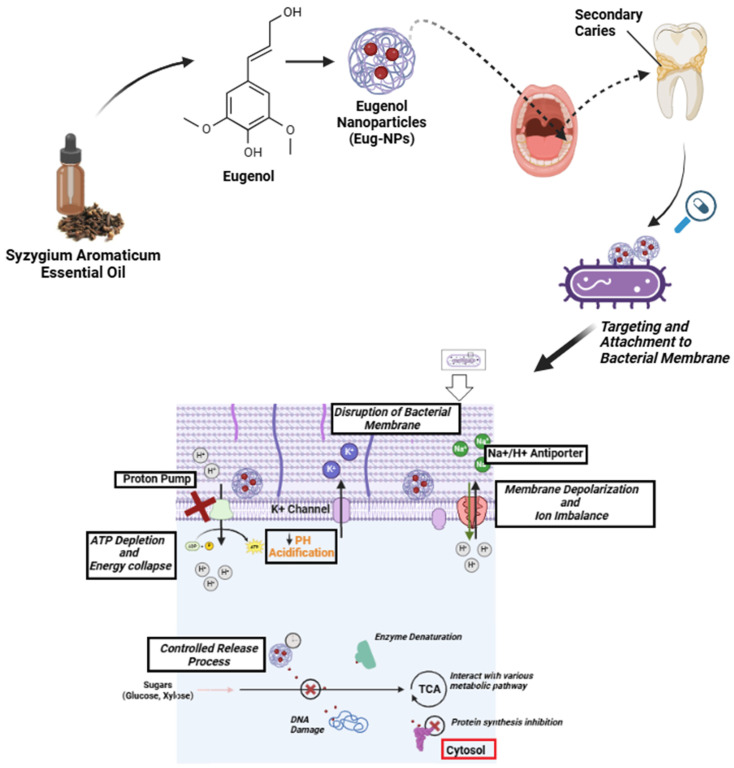
Schematic representation of eugenol-loaded nanoparticles in an antibacterial dental composite for controlled release.

Eugenol, chemically known as 4-allyl-2-methoxyphenol, is the main component of clove (*Syzygium aromaticum*) essential oil. It is a naturally occurring phenolic compound and makes up 45–90% of the composition of clove essential oil. In smaller concentrations, it is found in the essential oils of spices such as cinnamon, bay, tulsi, turmeric, and nutmeg [75]. This compound exhibits antimicrobial, antifungal, antioxidant, anticancer, anti-inflammatory, analgesic, repellent, and insecticidal effects [75,76]. In dental practice, eugenol has gained importance due to its biocompatibility with dental materials and efficacy against *S. mutans* [77,78], as well as other oral pathogens [77,79,80]. Eugenol acts by disrupting the microbial cell membranes, depleting intracellular ATP levels, and inducing membrane depolarization (Figure 1) [81]. Overall, it is a promising alternative to the conventional and synthetic antibiotics currently used in dentistry. In addition, eugenol can modulate the inflammatory responses of the dental pulp, reducing pain and sensitivity. Specifically, eugenol has been shown to inhibit cyclooxygenase enzymes and suppress the release of pro-inflammatory cytokines [82]. Altogether, these properties make eugenol a suitable candidate for long-term dental applications.

Although eugenol is associated with the above properties, its use is limited in dental applications. This is because eugenol is rapidly lost at room temperature. It also shows poor solubility in water. These properties of high volatility and hydrophobicity make the direct incorporation of eugenol into dental composites impractical [81]. Attempts to incorporate free eugenol in composite resins in the past have resulted in improper polymerization, which ultimately compromised their mechanical properties and overall material strength [83]. As a significant advancement in this area, eugenol is now modified into polymerizable derivatives before they are incorporated in dental materials. This process retains the bioactive properties of eugenol and improves its compatibility with dental materials. For instance, the copolymerization of the eugenyl methacrylate monomer with dopamine methacrylamide (a dental resin) produces composites that exhibit significant antibacterial activity against *E. coli*, *S. mutans*, *P. aeruginosa*, *L. monocytogenes*, *Bacillus pyocyaneus*, and *Salmonella typhimurium* [81]. Some advanced polymerization techniques are also reported in the literature for the stabilization of free eugenol. One such example is the conversion of eugenol into 4,4′-(butane-1,4-diyl)-bis(2-methoxyphenol) with the help of Ru-catalyzed olefin metathesis. This process produces high-performance polymeric materials with superior thermal stability and mechanical strength and good antibacterial activity [84]. Another example of a polymerization technique is the conversion of free eugenol into 4-allylpyrocatechol. This technique creates a light-curable polymer network with strong adhesion properties and long-term antibacterial activities [85]. An interesting study reported the synthesis of 19 eugenol derivatives with the help of esterification and addition reactions. All these derivatives showed promising antibacterial potential against *E. coli* and *S. aureus* [86]. Abdou et al. (2021) studied different processes to synthesize structural analogs of eugenol and reported a total of 176 possible structural modifications. Further, the study reported the improved antimicrobial and antioxidant properties of many of these analogs [87].

Besides the advantages of antibacterial and anti-inflammatory activities, the most advantageous application of polymerized eugenol-based materials is that it eliminates the need for toxic solvents. This environmentally friendly and sustainable agent has the potential to become a central, or at least a very important, part of modern dentistry practice [6,86,87].

### 4.2. The Integration of Nanoparticles

Reducing the dimensions of materials to their nanoscale changes their physico-chemical properties. In this range, the nano-materials exhibit quantum effects, altered electronic structures, and increased reactivity due to the high surface energy. They also interact better with other materials or surrounding environments due to the increased surface area to volume ratio [88]. Because of these characteristics, NPs are particularly useful in dental applications [89]. In restorative materials, they enhance strength, flexibility, and toughness, ultimately improving the mechanical properties of dental implants. More specifically, they help create strong adhesion to dental surfaces, increase wear resistance to withstand mechanical stress, and improve overall durability [90]. However, several key factors should be optimized before incorporating these nano-materials in dentistry applications. These mainly include uniform dispersion and stable inter-molecular interactions [88]. Once these parameters are met, NPs act as efficient carriers for bioactive materials which can be either encapsulated within NPs or integrated through other bonding mechanisms (such as hydrogen bonding, electrostatic interactions, or covalent attachments). Overall, these nano-systems allow for the controlled and sustained release of bioactive or therapeutic agents. Hence, NPs, in dental applications, can improve mechanisms such as antimicrobial and anti-inflammatory activities, re-mineralization processes, and drug delivery [74].

Integrating eugenol derivatives with suitable NPs can further improve the activity of dental composites. Individually, both NPs and eugenol derivatives can exhibit antibacterial activity and reduce the risk of biofilm formation and secondary infections. Both of these materials interact with bacterial membranes and increase their permeability, which causes the leakage of intracellular components [91]. Many NPs also catalyze ROS formation, inducing oxidative stress that damages proteins, lipids, and nucleic acids, and impairs the metabolic functions of microbial cells. However, the same can affect human cells and organs, and hence it is necessary to modify the NP surfaces optimally to reduce or prevent the cytotoxic effects associated with ROS generation [92]. Metal-based NPs prepared from silver release Ag+ ions that penetrate bacterial cells and disrupt their enzymatic activities. Similar action is exhibited by other metal NPs, including those prepared with gold, copper, zinc, titanium, gallium, aluminum, and platinum [93]. Studies also describe the notable antimicrobial properties of metal oxide NPs and carbon-based nano-materials, such as graphene oxide and carbon nanotubes [94,95]. The eugenol derivatives are also associated with these bioactive properties. When either eugenol or NPs are used individually in dental restoration models, factors such as sustained bioactivity cannot be accomplished. However, upon integration with NPs, a suitable model can be designed that can enable the controlled and sustained release of therapeutic agents such as eugenol. This controlled delivery mechanism will ensure prolonged antibacterial activity, maintain therapeutic concentration, and thereby minimize the need for re-interventions in restorative dentistry.

In the literature, a few studies have demonstrated the efficacy of polymeric NPs integrated with bioactive compounds, including eugenol. For example, a study incorporated zinc oxide NPs into zinc oxide eugenol cements [96,97]. This resulted in an improved polycrystalline structure and the dispersion of NPs in the cement matrix, which enhanced their mechanical properties compared to zinc oxide powder [98]. In another study, a polymeric nanocapsule was explored for the controlled delivery of eugenol. This nanocapsule efficiently encapsulated eugenol, maintained stability over an extended period, and reduced dental sensitivity [99]. A more advanced application of eugenol-integrated NPs was demonstrated by Sereda et al. (2023) with the development of acid-triggered release systems. These systems were programmed to release eugenol in response to the acidic environment caused by bacterial activity [100].

Free eugenol, commonly found in dental materials and in the eugenol-containing temporary cements, is known to inhibit the polymerization of resin-based cements, primarily due to its radical-scavenging activity [101,102,103]. It also leads to a reduction in resin-based materials’ bond strengths, particularly at higher concentrations. The eugenol inhibitory effect is significant within the first 24 h and up to 7 days after temporary cementation. After 14 days, the adverse effect on bond strength is no longer significant, suggesting that waiting at least 2 weeks after eugenol exposure can restore bonding effectiveness [104,105]. Eugenol presence in dentin or temporary restorative materials can compromise the immediate adhesion of resin cement and increase microleakage, especially at non-enamel margins. Some studies suggest that eugenol’s effects on bond strength may be less pronounced than is commonly believed [106]. The negative potential of eugenol contamination affects both etch-and-rinse and self-etch adhesive systems [105]. To surpass this issue, many strategies have been explored. One effective approach involves the use of polymerization accelerators, such as p-toluenesulfinic acid sodium salt, which can significantly increase the microtensile bond strength of adhesives to eugenol-contaminated dentin [107]. Furthermore, mechanical cleaning of the dentin surface and allowing sufficient time between temporary and permanent restorations may help restore bond strength [104,106]. In addition, another effective strategy is delaying the final cementation with resin-based materials by at least 14 days following the use of eugenol-containing temporary restorations [108,109]. This time lapse allows the inhibitory effects to subside naturally, restoring the bond strength to baseline levels.

Another emerging solution involves the encapsulation or immobilization of eugenol or its derivatives within the cement matrix [110,111]. The encapsulation method leads to the isolation of eugenol physically during the polymerization process and allows for its controlled release afterward, thus preserving its therapeutic benefits without compromising the polymerization or mechanical integrity of the material [81,112,113]. Through eugenol encapsulating in nanoparticles made of polymeric materials such as chitosan, zein, or PLGA, the potential negative impact on resin polymerization can be minimized, and the therapeutic benefits of eugenol (like its antimicrobial and anti-inflammatory properties) can be delivered post-polymerization [1,81,114]. This allows for eugenol’s therapeutic advantages, without compromising the mechanical properties of the restorative material or affecting the adhesion quality. In addition, encapsulated eugenol can be released in a controlled manner over time, ensuring that its antimicrobial and anti-inflammatory effects are sustained long after the initial polymerization phase, which is beneficial in preventing secondary caries and promoting healing around the restoration area without affecting the polymerization of resins [110,115,116]. It can also offer a strategic advantage through allowing the controlled and delayed release of active compounds [117]. This encapsulation could prevent the interference between eugenol and resin-based cement polymerization during the critical curing phase, effectively mimicking delaying the final cementation benefits. While traditional protocols rely on waiting for residual free eugenol to dissipate naturally over time, typically over 14 days, nano-encapsulation gives clinicians control over when eugenol is released.

From a clinical standpoint, eugenol-loaded nanoparticles appear to offer promising benefits in enhancing dental restorative materials, particularly in cases where antimicrobial and anti-inflammatory properties are essential [96]. These nanoparticles can provide sustained antimicrobial activity and help minimize post-operative inflammation while reducing the risk of interference with resin adhesion [118]. Such properties are especially advantageous in restorative treatments for high-risk patient groups, such as those with a history of dental caries or periodontal disease, potentially leading to improved clinical outcomes.

### 4.3. Regulated Release Process

The controlled and sustained release of antimicrobial or other bioactive agents is a critical strategy for enhancing dental material efficacy and longevity. One of the most promising approaches is the embedding of NPs into dental composite resins to ensure a prolonged therapeutic effect. The bioactive material effectiveness with NPs depends on their uniform dispersion and stability, which directly influence release kinetics. In the literature, various mechanisms have been described to optimize the regulated release of agents. These include diffusion-controlled, degradation-mediated, and stimuli-responsive approaches [81]. By refining these strategies, it is possible to develop next-generation dental composites that can overcome the present limitations and provide improved antibacterial protection, biofilm inhibition and re-mineralization capabilities.

In diffusion-controlled release kinetics, the bioactive materials diffuse through the polymer matrix at a rate that is influenced by factors such as polymer porosity, NP size, and resin hydrophobicity. Similarly, in biodegradable polymeric NPs, the breakdown of the polymer matrix gradually releases the encapsulated bioactive agent over time. The latter mechanism is particularly useful for long-term therapeutic applications. Basically, these processes follow a Fickian diffusion model, where the release rate is proportional to the concentration gradient [119]. Briefly, the polymeric NPs form stable matrices that encapsulate the bioactive agents and release them gradually through diffusion or polymer degradation. For example, polylactic acid and poly lactic-co-glycolic acid NPs provide controlled degradation, extending the antimicrobial effect of bioactive agents for weeks or months [120]. Another technique known as Layer-by-Layer (LbL) assembly is used for the slow, stepwise release of antimicrobial agents, significantly prolonging their therapeutic effect. In this technique, alternating layers of charged NPs and bioactive molecules are deposited on composite fillers, commonly made from silica or zirconia. They are most commonly used for the re-mineralization process and incorporate silver or calcium phosphate ions [121].

Furthermore, many properties of nano-carriers are material specific. For instance, polymeric nanocarriers such as zein, sodium caseinate, and pectin-based colloidal nanoparticles have shown a high encapsulation efficiency and form ~140 nm-sized particles [122]. Silica-based nano-carriers are known for their high stability compared to metal-based NPs. Surface-functionalized NPs, such as silica-coated or polymer-grafted particles, improve dispersion and adhesion within the composite resin [123]. The crystallization temperature of lipid-based carriers affects the encapsulated bioactive compound release rate [124]. The addition of cloisite 5A nanoclay in zinc oxide eugenol cements modulates the diffusion rate and further enhances the material’s antibacterial properties [98].

An advanced, regulated-release system is also reported in the literature which utilizes stimuli-responsive NPs to release bioactive agents in response to specific environmental triggers such as pH, temperature, and enzymatic activity. This system is developed while keeping in mind the highly dynamic nature of the oral environment that continuously undergoes pH fluctuations due to dietary intake, bacterial metabolism, and saliva composition. For instance, the chitosan–guar gum and chitosan–pectin nanocapsules have been shown to exhibit pH-sensitive release kinetics. The release rates of these nanocapsules range from 30% at acidic pH (3) to 80% at neutral pH (7.4) over three hours. This allows for enhanced antibacterial activity in acidic environments, such as those created by bacterial biofilms [100]. Similarly, casein-coated calcium carbonate microspheres have been developed as pH-responsive carriers [125]. Temperature-sensitive nanoparticles provide another control layer by releasing their bioactive contents when exposed to elevated temperatures, such as those induced by local inflammation or bacterial infections [126].

### 4.4. Dual Functionality

The present review supports the view that the integration of eugenol derivatives and NPs into dental composites will be a significant advancement in restorative dentistry. Eugenol exhibits broad-spectrum antibacterial activity that can prevent secondary caries and biofilm formation [116,127,128]. In addition, eugenol can mitigate the inflammatory response following dental procedures, such as fillings, root canals, or implant placements, which leads to post-operative sensitivity and discomfort [81]. Among the various benefits of eugenol and NPs highlighted throughout this review, this dual functionality of the antibacterial and anti-inflammatory efficacy of this combination is of prime significance. Overall, these properties are ideal since they overcome the two main concerns and limitations of current restorative procedures. Additionally, embedding eugenol derivatives in NPs ensures the optimization and maintenance of release kinetics that will allow for the gradual and sustained release of the antimicrobial agents over time.

## 5. Advantages and Potential Applications

### 5.1. Clinical Advantages

The clinical advantages of integrating eugenol derivatives with NPs can be summarized as follows [87,89,93,98,99]:

Since conventional dental composites do not inherently possess antimicrobial properties, they are vulnerable to bacterial colonization and biofilm formation. The suggested eugenol and NP model in dental composites will primarily be able to prevent secondary caries, which is a major cause of restoration failures.

1. Eugenol NPs exhibit a broad spectrum of antimicrobial properties, not only against *S. mutans* but also against a range of oral pathogens. Hence, the suggested model will improve the overall oral health of individuals undergoing restorative procedures.
2. The flaws in the design of dental restorative materials leading to micro-cracks and leakages can be overcome with the help of this model, where NPs can form protective barriers and eugenol can further reduce the risk of microbial infiltration around restorations. Eugenol’s antimicrobial action can also minimize dental restoration material degradation and enhance their structural integrity.
3. The sustained antimicrobial action of NPs incorporating eugenol can ensure controlled and prolonged release, ensuring continuous antibacterial protection. The sustained release mechanisms will also help in minimizing the fluoride dependence that is used in traditional composites to prevent secondary caries.
4. Also, these models will not require frequent reapplication, which will decrease the associated treatment cost and improve patient comfort.
5. A significant decrease in post-operative sensitivity and inflammation can be expected with the use of this model due to the anti-inflammatory, analgesic, and wound healing properties associated with eugenol. Eugenol can desensitize nerve endings and hence prevent post-procedure discomfort and dentin hypersensitivity. It also reduces oxidative stress and enhances fibroblast activity, both of which support tissue regeneration and promote recovery.

### 5.2. Environmental and Biocompatibility Benefits

In addition to clinical advantages, the use of eugenol with an appropriate nano-carrier is a sustainable approach. The benefits of this approach can be summarized as follows [88,92,93,97,99,100]:

➢ Unlike the synthetic antimicrobial agents used in medicinal applications, eugenol is biocompatible, which reduces the risk of cytotoxicity. Encapsulation techniques further minimize the cytotoxicity to human cells while maintaining efficacy against pathogenic bacteria.
➢ The mechanism of action of eugenol on bacterial cells is by the disruption of membranes rather than by affecting DNA. This lowers the likelihood of oral pathogens developing antimicrobial resistance.
➢ Eugenol nanoparticles can be bio-fabricated using plant extracts. The incorporation of biodegradable polymer carriers such as chitosan, PLA, or PCL further encourages the potential for green synthesis, which helps in reducing the environmental impact of medical waste.

### 5.3. Wider Applications

In traditional practices, eugenol has been widely used for its antibacterial activity. This review mainly describes the benefits associated with the antibacterial and anti-inflammatory potential of eugenol and NPs. In the absence of NPs, the rapid release of eugenol leads to short-lived activities [100]. By incorporating eugenol NPs in the dental models, a sustained release mechanism will be ensured to prevent infections for an extended period. Beyond these applications in permanent restoration processes, eugenol NPs offer a much wider potential due to their wound healing properties and mild anesthetic effects that can be positively exploited in dental practices. A few possibilities are summarized below:

1. The temporary restoration materials such as zinc oxide cements can be mixed with eugenol NPs and applied before permanent restorations are placed in the oral cavity. Similarly, they can be used in endodontic sealers used in root canal treatments [93]. Moreover, like amalgam and composite restorations, improper installation of orthodontic appliances such as brackets, aligners, and retainers can lead to bacterial accumulation [89]. Hence, eugenol NPs will prove to be extremely useful.
2. Unlike the common cases of secondary caries caused by *S. mutans*, the elderly population is more susceptible to fungal infections caused by Candida albicans. Similarly, immune-compromised individuals are at increased risk of oral infections caused by uncommon pathogens [92]. The broad-spectrum activity of eugenol NPs can be extremely beneficial under these circumstances.
3. The traditional fluoride-based sealants can be mixed with eugenol NPs. This approach will extend the antibacterial and anti-inflammatory property model to one which entails an overall dental package by additionally preventing demineralization due to metabolic acids [87].
4. The common dental varnish side effects, especially those containing fluorides, include temporary discoloration, a burning sensation, tooth sensitivity, and gum irritation. The eugenol NPs’ anesthetic, soothing, antimicrobial, and anti-inflammatory effects may prove helpful in preventing these side effects [87].
5. The eugenol NPs also have the potential to replace traditional irrigation solutions such as chlorhexidine or sodium hypochlorite, which are associated with tissue irritation or cytotoxic effects [94].

## 6. Challenges and Future Directions

### 6.1. Challenges

The approach of using dental composites containing eugenol-integrated NPs is highly promising in the field of dentistry. However, we are required to address several challenges before this approach becomes widely appreciated and commonly used. Overall, these challenges can be summarized as follows [100,129,130]:

It is required to ensure the long-term stability of eugenol-containing NPs that are incorporated in the dental composite matrices. This can be achieved by optimizing the encapsulation techniques, selecting appropriate polymeric carriers and confirming the compatibility of bioactive agents with dental materials.

The optimization of other key factors such as polymerization conditions (temperature, photoinitiators), NP dispersion (preventing aggregation), and chemical interactions is also necessary to maintain the stability of bioactive agents, NPs, as well as dental materials.

It is equally necessary to design a model that can ensure the sustained release of eugenol over extended periods. The ideal model should be able to sense pH variability in the oral cavity due to saliva and enzyme activity.

Lastly, the polymeric nano-carriers selected should not undergo rapid biodegradation, since it can compromise the structural integrity of the composite.

Also, although eugenol is generally recognized as safe (GRAS), there is a need to ensure that its nano-particulate form is safe to use, since it is well known that the properties of compounds change upon conversion to nano-dimensions. Hence, polymerized and stabilized eugenol integrated with NPs should be evaluated for toxicity towards oral tissues over short- and long-term exposure. Studies should also evaluate neurotoxicity, allergic reactions, and systemic absorption through oral mucosa. However, these studies are extremely challenging due to the absence of standardized protocols for evaluating the efficacy of this newly designed model system of dental composites. In general, the scaling and production of uniformly dispersed NPs encapsulating a bioactive material is a significant challenge in medicinal applications due to their high costs. Yet, long-term in vivo studies will be necessary to prove the effectiveness of the suggested model in the oral environment, which will be both costly and time-consuming [130,131].

### 6.2. Future Research Directions

Presently, there are limited studies published in the online database that have used dental restorative materials containing eugenol encapsulated in NPs. These studies have overall described both the challenges and potential of this model in preventing microbial colonization, reducing inflammation, and enhancing patient comfort. Based on these studies, the key aspects that require further investigation to fully optimize their effectiveness can be summarized as follows [100,129,130]:

1. Firstly, as described before in this review, the NP synthesis methods should be optimized to improve factors such as the stability, bioavailability, and sustained release kinetics of eugenol. One of the approaches to achieve these parameters is to employ eco-friendly synthesis methods that utilize plant-derived biopolymers or bio-fabrication techniques.
2. Research is also necessary to optimize extended-release functions in the suggested model that can prevent oxidative damage. This can be achieved by using core–shell structures or layered NPs to encapsulate polymerized and stabilized eugenol.
3. Further research also requires much focus on improving the dispersion of NPs in the composite matrices to prevent the premature leaching of bioactive agents like eugenol. This can be achieved by modifying NP surfaces with biocompatible coatings made of silica, chitosan, polymeric shells, or other biocompatible materials.

Most importantly, future studies should focus on developing pH- and enzyme-sensitive NPs that can release eugenol in response to changes in the oral environment. This is necessary to optimize the therapeutic efficacy of the suggested model.

In addition, currently, most studies have demonstrated the antibacterial potential of eugenol containing dental restoration models with in vitro studies. Future studies should focus on in vivo methods to evaluate the performance of stabilized eugenol in NPs in oral environments. Specifically, more studies should be designed to study the conditions of the oral system by mimicking saliva flow, bacterial colonization, and mechanical stress. These conditions may be integrated with biofilm models to closely improve the assessments of factors in the naturally occurring oral environment that lead to secondary caries [132]. Similarly, animal studies and longitudinal studies are necessary for evaluating NP retention, bioactivity, and systemic safety over extended periods. Through these measures, it is also possible to gather enough evidence that will eventually help in developing standard regulatory protocols for NP- and eugenol-based dental materials in the future.

Another important and practical directive for future studies is to concentrate on re-mineralizing agents such as calcium, fluoride, or phosphate ions. Some of the literary evidence of these approaches is available presently. For instance, Anil et al. (2022) reviewed the potential of nano-hydroxyapatite or amorphous calcium phosphate to promote enamel re-mineralization [133]. Data suggest that it is possible to develop multifunctional dental composites that can combine re-mineralizing agents, NPs, and bioactive agents (such as eugenol) to prevent secondary caries recurrence and reduce inflammation. Similarly, studies have suggested the potential of intra-oral fluoride release mechanisms to enhance cariostatic effects and prevent demineralization at the same time [131,134]. Combining these properties with polymerized and stabilized eugenol derivatives integrated with NPs can further discourage cavity formation and the occurrence of oral diseases. Precisely, these examples prompt us to consider designing dual-phase nanocarriers. These can be programmed to release bioactive agents like eugenol upon installation in the oral cavity to ensure antibacterial action and the prevention of infections. This action can be followed by re-mineralizing strategies with the help of mineral ions to restore the affected enamel. For instance, a detailed review by Montoya et al. (2023) suggested the benefits of silica-based bioactive glass NPs doped with calcium and phosphates compared to other known smart dental models [74]. We may further enhance this model by adding stabilized eugenol in NPs to facilitate antibacterial, anti-inflammatory, as well as re-mineralizing actions. Altogether, these attempts in future studies may lead to the design of next-generation therapeutic composites that will successfully prevent secondary caries and support long-term oral health [74,131].

Despite the comprehensive scope of the studies and the advancing promise of eugenol-integrated nanoparticle dental composites, a few shortcomings need to be highlighted. Firstly, many in vitro and some in vivo studies support the antimicrobial and anti-inflammatory activities of eugenol; however, clinical support is limited. Most of the current evidence stems from laboratory models which offer the limited reproduction of the oral cavity’s intricate system dynamics, such as the variability in saliva, pH changes, and diverse biofilms encountered in patients.

Secondly, though the nano-encapsulation offers better stability and the controlled release of eugenol, the long-term consequences of these nanoparticles on oral tissues and systemic health are largely unknown. Worrisome aspects arise from scarce toxicological data on the chronic exposure and biodegradability of some nano-carriers regarding their potential accumulation or deleterious biological interactions.

Third, the mechanical properties of eugenol-loaded composites, particularly in relation to fracture resistance, wear, and bonding strength, are still under investigation. Bioactive agents’ inclusion into dental materials may compromise their mechanical integrity, and striking a balance between bioactivity and physical durability remains a significant challenge.

These limitations further point to the necessity of establishing uniform experimental protocols, and well-designed clinical trials to determine the long-term efficacy, safety, and practicability of eugenol–NP in real-world dental practice.

## 7. Conclusions

The dental restoration development model containing stabilized eugenol in NPs represents a significant restorative dentistry innovation. In addition to traditional dental composites’ benefits, this model can address their microbial contamination limitations and post-operative inflammation. Due to these limitations, the traditional dental materials are associated with biofilm formation, secondary caries, and prolonged healing times. By incorporating eugenol-integrated NPs, the dental materials will be able to provide protection against *S. mutans* and other oral pathogens as well as provide anti-inflammatory effects. Through this approach, the therapeutic dental restoration methods can have the potential to revolutionize the restorative dentistry by making them more effective, biocompatible, and patient-friendly. In addition, the controlled release of eugenol over time will ensure prolonged efficacy. This can substantially reduce the need for additional treatments. Most importantly, the use of the NP encapsulation method overcomes the limitations of free eugenol, such as volatility and poor water solubility. This approach can hence make the suggested model highly adaptable and promising for modern dental applications.

Furthermore, this review highlights that the innovation lies not only in the bioactive compound inclusion like eugenol, but in the strategic engineering of its delivery through nano-encapsulation. This allows clinicians to benefit from the therapeutic properties of eugenol without compromising polymerization or mechanical integrity, and introduces a level of control over its release that can be tailored to clinical needs. This dual advantage suggests the approach has strong clinical relevance, especially for high-risk patients where both antimicrobial protection and improved healing are essential.

Like any new medicinal approach, further research and development will be required for optimizing NP formulations, enhancing mechanical strength, and ensuring the long-term clinical performance of the NP-stabilized eugenol model suggested in this review. This can only be achieved through large-scale clinical trials that will truly validate the safety, effectiveness, and patient outcomes of using this model. Only through these steps and continued advancements will eugenol NP-based composites have the potential to set a new standard in restorative dentistry and become a widely used approach for improving oral health outcomes and patient care worldwide.

## Data Availability

No new data were generated or analyzed in this study. Data sharing is not applicable to this article.
